# A Robust Wireless Sensor Network Localization Algorithm in Mixed LOS/NLOS Scenario

**DOI:** 10.3390/s150923536

**Published:** 2015-09-16

**Authors:** Bing Li, Wei Cui, Bin Wang

**Affiliations:** College of Information Science and Engineering, Northeastern University, 110819 Shenyang, China; E-Mails: rose.neu2011@aliyun.com (W.C.); binwang@mail.neu.edu.cn (B.W.)

**Keywords:** Gaussian mixed model, multidimensional scaling, wireless sensor network, RSSI

## Abstract

Localization algorithms based on received signal strength indication (RSSI) are widely used in the field of target localization due to its advantages of convenient application and independent from hardware devices. Unfortunately, the RSSI values are susceptible to fluctuate under the influence of non-line-of-sight (NLOS) in indoor space. Existing algorithms often produce unreliable estimated distances, leading to low accuracy and low effectiveness in indoor target localization. Moreover, these approaches require extra prior knowledge about the propagation model. As such, we focus on the problem of localization in mixed LOS/NLOS scenario and propose a novel localization algorithm: Gaussian mixed model based non-metric Multidimensional (GMDS). In GMDS, the RSSI is estimated using a Gaussian mixed model (GMM). The dissimilarity matrix is built to generate relative coordinates of nodes by a multi-dimensional scaling (MDS) approach. Finally, based on the anchor nodes’ actual coordinates and target’s relative coordinates, the target’s actual coordinates can be computed via coordinate transformation. Our algorithm could perform localization estimation well without being provided with prior knowledge. The experimental verification shows that GMDS effectively reduces NLOS error and is of higher accuracy in indoor mixed LOS/NLOS localization and still remains effective when we extend single NLOS to multiple NLOS.

## 1. Introduction

Node localization is an essential technique in order to guarantee high-effectiveness of Wireless Sensors Network (WSN). In the indoor space, when the wirelessly propagated Line-of-Sight (LOS) paths between the target node and the anchor node are obstructed by obstacles (like people, table, wall, *etc.*), the radio waves must be propagated via the indirect paths such as refraction and reflection, which are referred to as Non-Line of Sight (NLOS) paths. Comparing with the LOS scenario, the NLOS scenario contains a positive excess distance measurements delay, which is called NLOS error. Unfortunately, traditional methods of node localization in LOS environment are hard to yield accurate Maximum Likelihood (ML) and often generate very noisy and unreliable final estimation results due to NLOS error. Therefore, we believe a new method is necessary to improve the accuracy and usability of NLOS node localization. In this paper, we present an efficient, accurate and scalable method for indoor mixed LOS/NLOS node localization that aim to reduce the NLOS error within NLOS propagation in the real environment.

In recent years, several attempts have been made to reduce the NLOS error in node localization. The general process of these attempts of NLOS localization mainly includes NLOS error identification and NLOS error elimination two steps. By NLOS error identification, one refers to find nodes that are in an NLOS environment or rebuild a LOS propagation model in NLOS scenario [1]. Kalman filtering was also used to reconstruct NLOS signals [2]. Residual test was used to identify NLOS error, and then localization can be realized simply via LOS measurements [3]. The above algorithms need the acquisition of signal statistical properties, but such prior knowledge is hard to access in the real environment.

As for NLOS error elimination, the weighted least squares estimation based on Taylor series expansion (TS-LS) is widely used, but TS-LS needs pre-defined weights, which are usually obtained by a complex statistic model [4]. Another method is a residual weighting approach, which is used to alleviate the NLOS error when it is very unmeasurable within the measurement range [5]. The select residual weighting (SRwgh) localization algorithm based on classical NLOS error suppression algorithms aims to reduce the computation complexity and simplify the interference in NLOS [6]. An ML function was used to elicit a ML estimation algorithm that contained the closed-form solution [7]. Mcguir considers an NLOS scenario and proposed a non-parametric and highly efficient approximate localization algorithm based on a time difference of arrival [8]. A hidden Markov model was applied to localization [9]. The filtering method is proposed in [10,11] to reduce the NLOS error. Kalman or extended Kalman filtering was used in localization, and its main principle is to add new information and reduce error along with time prolonging [12]. This precise algorithm can be applied to track a moving target in non-stationary random processes, but it requires a specific localization parameter at varying time points, and become non-convergent in some situations [12]. The scattering of wave beam in an NLOS scenario is used to build a propagation model [13]. In a co-localization algorithm, the neighbor node cooperates to reduce the interference of NLOS [14]. A NLOS error-reducing localization algorithm based on constraint conditions was proposed, including some more accurate criteria compared with CRLB [15]. An interactive multi-model (IMM) is built for NLOS localization, but it requires *priori* information [16].

In recent years, a lot of novel ideas and solutions have emerged for WSN localization. The localization technique based on multidimensional scaling (MDS), which first proposed by Shang *et al.* [17], offers a new solution of node localization. The MDS method that concerns dimension reduction and feature extraction is widely used to do data analysis and data visualization in fields of physics, biology and psychological phenomenon research, as well as pattern recognition, machine learning and so forth [18,19,20]. Comparing with previous localization algorithms, the MDS-based localization algorithms can simultaneously locate multiple nodes by utilizing the associated information among all nodes within the network. The advantages of MDS are that, one can obtain actual positions between nodes by setting only a few anchor nodes, besides, the anchor nodes’ deployment has no strict restriction. Moreover, when anchor nodes are unavailable, one can still get relative positions between nodes. Recently, a variety of localization methods based on classical MDS method have been applied to sensor network [21,22] as well as cellular network [23].

Existing algorithms in wireless sensor network localization can be broadly divided into two classes: range-based and range-free. The class of range-based algorithms is required to provide the accurate distance estimation between the target node and anchor node. Moreover, range-based algorithms require each sensor node to be equipped with a more powerful CPU. There are several approaches to get the distance estimation: received signal strength indicator (RSSI) [24], time of arrival (TOA), and time different of arrival (TDOA) [25]. The class of range-free algorithms can reduce the energy consumption and the demands for special hardware, but their accuracy is lower than the former one, such as Approximate Point-in-Triangulation Test (APIT) [26] and Distance Vector-Hop (DV-Hop) algorithms. The existing NLOS localization algorithms are generally based on the distance measurements from some known nodes and localize an unknown node according to the distances relations between them. A common localization mechanism used by these algorithms is RSSI due to its low-cost, no extra hardware consumption, and simple computation, so RSSI gets wide application in node distance measurement. Based on the signal intensity measured from a base station, a localization system first computes the distance between a node and the base station by a signal propagation model, and then computes the position of that node by a localization algorithm. In the indoor space, however, RSSI is easy to be disturbed by shadow fading, multipath effect and NLOS, therefore, RSSI-based distance measurements often contain large errors and leads to final result inconsistency. Hence, RSSI-based distance-measurement and localization algorithms do not perform well in practical indoor NLOS applications. Moreover, it is computation-consuming for statistical data transformation when the parameters of wireless signal propagation model change as the environment dynamically changes. To solve these shortcomings, in this paper, we present an indoor Gaussian mixed model based non-metric Multidimensional (GMDS) localization algorithm. First, we model the RSSI measurement data for estimating RSSI actual value that is closest to that of the LOS scenario based on the NLOS error’s features by using a Gaussian mixed model (GMM). Second, we apply an improved MDS to indoor NLOS localization. In MDS, we use estimated wireless signal intensity values to localize position directly, which alleviates the errors and reduces the computation in transforming RSSI data to distances that happened in traditional signal propagation model. Simulation and practical application results demonstrate that GMDS is feasible and effective to indoor NLOS scenario with high robustness and precision, satisfying most indoor localization applications.

This paper is organized as follows. Section 2 briefly describes the distance measurement models. Section 3 presents the GMM and its application in RSSI estimation. Section 4 describes the RSSI based GMDS method. Section 5 shows the experimental results highlighting the performance of our algorithms. Section 6 concludes this paper.

## 2. Distance Measurement Models

### 2.1. RSSI-Based Distance Measurement Model

The RSSI localization mechanism is that estimates of transmitter-receiver distance via taking signal intensity into a known channel fading model. Since the signal will gradually fade and the intensity will decrease with propagation distance extending, the finally received signal intensity can be used to estimates the transmitter-receiver distance. Generally, the signal propagation model is expressed as [27]:

(1) $$\left[RSSI(d)=P_{T}-PL(d_{0})-10\eta \mathrm{lg}(d/d_{0})\right]$$

Where *RSSI*(*d*) is the signal intensity received by the node at distance *d*; *P_T_* is the transmitted signal intensity; η is a scale factor between path length and path loss; *PL*(*d*_0_) is the signal intensity received at the reference distance *d*_0_, which can be obtained empirically or from hardware specifications.

### 2.2. RSSI Measurement Error Model

Based on the above RSSI-based distance measurement model, we propose a RSSI measurement error model as follows [27]:

(2) $$\left[{\hat{P}}_{r}=\left\{ \begin{array}{l} P_{r}+m_{los}^{F}+N_{los},\mathrm{LOS}\\ P_{r}+m_{nlos}^{F,O}+N_{nlos},\text{NLOS} \end{array}\right.\right]$$

where ${\hat{P}}_{r}$ is the receiver-transmitter RSSI measurement; $P_{r}$ is the signal intensity in the case of LOS; $m_{\text{los}}^{F}$ is the error due to multipath effect in LOS scenario; *F* is the radio frequency-caused error; $m_{\mathrm{nlos}}^{F,O}$ is the NLOS error; *O* is the obstacle-caused error. Measurement-caused error will occur in both LOS and NLOS scenarios, expressed as $N_{los}$ and $N_{nlos}$ respectively. Measurement error is the noise caused by the experimental devices or the operators, generally, it is far smaller than NLOS error, but $m_{\mathrm{nlos}}^{F,O}$ is interfered by obstacles, it is far larger than $m_{\mathrm{los}}^{F}$. Therefore, it has:

(3) $$\left[\left|N_{\text{los}}\right|,\left|N_{\text{nlos}}\right|\leq \left|m_{\text{los}}^{F}\right|\leq \left|m_{\text{nlos}}^{F,O}\right|\right]$$

Therefore, we mainly discuss the methods for eliminating $m_{\mathrm{nlos}}^{F,O}$ in NLOS scenario.

## 3. GMM and Its Application in RSSI Estimation

Observing that RSSI measurement contains bias in NLOS compares to LOS (namely, NLOS error) due to obstacle’s impact, one shall not expect a direct computation model as LOS does. From the technical perspective, we believe that RSSI measurements in both LOS and NLOS scenarios follow Gaussian distribution [19,20]. Thus, we used GMM as underlying distribution of RSSI measurements. The estimated values of RSSI in LOS and NLOS scenarios can be expressed as Gaussian submodels by different probability distributions in GMM.

### 3.1. About GMM

In this subsection, we start with a brief review of GMM. Let $X=\left\{x_{1},x_{2},\ldots x_{N}\right\}$ denotes the vector of the *N*-dimension of RSSI estimated values both in LOS and NLOS scenarios. The probability density function of RSSI estimated values in the LOS scenario satisfies $f_{\mathrm{los}}\left(x\right)\sim N\left(P_{r\_\mathrm{los}}+m_{\mathrm{los}}^{F},\sigma_{\mathrm{los}}^{2}\right)$, where $P_{r\_los}$ is the RSSI value in LOS scenario; $m_{los}^{F}$ is the fixed value under stable indoor environment, this value can be set to 0 if there is no large deviation; $\sigma_{los}^{2}$ is the variance in LOS scenario. The RSSI estimated values in NLOS scenario follow Gaussian distribution: $f_{nlos}\left(x\right)\sim N\left(P_{r\_nlos},\sigma_{nlos}^{2}\right)$, where $P_{r\_nlos}=P_{r\_los}+m_{nlos}^{F}$. Thereby, we obtained the following K-order GMM probability density function:

(4) $$\left[f\left(x;\theta \right)=w_{1}f_{los}+\sum_{k=2}^{K}w_{k}f_{nlos,k}=\sum_{k=1}^{K}w_{k}g\left(x;P_{k},\sigma_{k}\right)\right]$$

where $g\left(x;P_{k},\sigma_{k}\right)=\frac{1}{\sqrt{2\pi }\sigma_{k}}{\text{e}}^{-\frac{1}{2}{\left(\frac{x-P_{k}}{\sigma_{k}}\right)}^{2}}$ is the *N*-dimension joint Gaussian probability distribution of each sub-distribution; $w_{k}$ is a mixed weight and satisfies the constraint $\sum_{k=1}^{K}w_{k}=1$; $\sigma_{k}$ is the covariance matrix; $P_{k}$ is the mean vector of each GMM, it denotes the RSSI values between the target node and the anchor node. Generally, a complete GMM involves covariance matrix, parameter mean vector, and the mixed weights, and it can be expressed as $\theta =\left(\theta_{1},\theta_{2},\ldots \theta_{K}\right)=\left(\left(w_{1},P_{1},\sigma_{1}\right),\left(w_{2},P_{2},\sigma_{2}\right),\ldots ,\left(w_{K},P_{K},\sigma_{K}\right)\right)$, where *K* denotes the total number of different error distributions, including LOS error, and varying intensity of NLOS error. With the number of data increasing, the probability density function of NLOS error trend to be smooth, and thus a limited number of Gaussian density functions are enough to smoothly approach the density function of measurements. Usually, a probability density function can be built by properly choosing GMM components and appropriately setting the means, covariances and mixed weights.

As the above section suggested, NLOS error is a positive value according to distance, and based on the signal fading model, the real RSSI value between receiver and transmitter can be approximated to the maximum among all RSSI estimated values.

### 3.2. Estimation of GMM Parameters

To express the RSSI measurements vector by GMM, one shall classifies the eigenvectors, the probability density function of each category can be regarded as an instance of Gaussian distribution, and the centers of each category are the mean value of corresponding Gaussian distribution instance, and sets the covariance matrix as its discrete degree.

The process of estimating GMM’s parameters is computing model parameters by a given set of measurements along with certain criteria, and thereby making evaluated probability distribution optimally satisfies the estimated RSSI values. A common parameter estimation method is maximum likelihood estimation (MLE), which sets a group of training vector series of estimated RSSI values: $X=\{x_{1},x_{2},\ldots ,x_{N}\}$.

The likelihood of GMM is expressed as:

(5) $$\left[F\left(x;\theta \right)=\prod_{k=1}^{K}w_{k}g\left(x;P_{k},\sigma_{k}\right)\right]$$

The goal of training is to find a group of parameter $\theta^{*}$ that maximize F(x;θ):

(6) $$\left[\theta^{*}=\arg_{\theta }\max F\left(x;\theta \right)\right]$$

Equation (6) is a nonlinear function about θ, so exhaustively computes θ that maximizes F(x;θ) is ineffective. The expectation maximization (EM) algorithm is often used to perform effective parameter estimation as a substitute of MLE algorithm in practice. EM is a recurring ML algorithm and used to train data series to estimate model parameters. The computation of EM starts from a predefined initial value of θ, then iteratively updates the next new parameter based on the equation, that makes likelihood of the new parameter satisfies $F\left(x;\theta^{*}\right)\geq F\left(x;\theta \right)$. The new parameter of current round is regarded as initial value for next round training, and continuously running iterative operation until converging. The re-estimated mixed weights, means and variances at round *k* as follows:

(1) The re-estimated probability weight is:
  (7) $$\left[w_{k}=\frac{1}{N}\sum_{n=1}^{N}w\left(k|n\right)\right]$$
(2) The re-estimated mean is:
  (8) $$\left[P_{k}=\frac{\sum_{n=1}^{N}w\left(k|n\right)x_{n}}{\sum_{n=1}^{N}w\left(k|n\right)}\right]$$
(3) The re-estimated variance is:
  (9) $$\left[\sigma_{k}=\sqrt{\frac{\sum_{n=1}^{N}w\left(k|n\right){\left(x_{n}-P_{r,n}\right)}^{2}}{\sum_{n=1}^{N}w\left(k|n\right)}}\right]$$
  where w(k|n) is posteriori probability and can be expressed as:
  (10) $$\left[w\left(k|n\right)=\frac{w_{k}g\left(x_{n};P_{k},\sigma_{k}\right)}{\sum_{i=0}^{K}w_{i}g\left(x_{n};P_{i},\sigma_{i}\right)}\right]$$

In the above equation, the computation of expectation (step E) and maximization (step M) in EM algorithm run synchronously and iteratively update steps E and M by computing the equation. The iteration ends when the likelihood function is maximized. EM algorithm performs well on solving the problem of GMM parameters estimation using MLE.

### 3.3. Clustering and Initialization

The above analyses show that GMM parameters can be estimated using EM algorithm. Since the EM algorithm is easy to fall into a local maximum, so the initialization of parameter becomes an important issue. The initial parameters of GMM include mixed coefficient, mean vector, covariance matrix, and the number of categories. The number of components *K* is usually set by user’s experience, in this paper, *K* value is set by the following steps: firstly, we carry out simulation analysis in MATLAB by setting 10 kinds of non-line-of-sight error, that is, *K* = 1, 2, ..., 10; then, in each kind of non-line-of-sight situation, we run GMM 10 times and analyze the deviation between the estimated RSSI value and true value under such situations. The experiment result shows that we can obtain an acceptable result which close to the optimal results if we set *K = 2*. Even though we can get a better result when setting a larger *K* value, however, the computational complexity increases drastically with the increasing value of *K*. Finally, we choose *K = 2* from the view of trade-off between computational complexity and a better result.

Usually, EM parameters can be initialized in two ways: (1) randomly select several vectors from the training data as the initial parameters; (2) approximately estimate the sample distribution by using clustering, and based on this, set the mean as the initial value to get a better initial value. We simply set the covariance matrix as a diagonal matrix.

The clustering is grouping eigenvectors to specific categories, and then the means and covariance of each category are computed and used as the initial values. A weight is defined as the proportion of eigenvectors in total eigenvectors of a category. In practice, *K*-means clustering is commonly used. It aims to group *n* samples into *K* categories, so that the samples in the same category have maximal similarity. In other words, it minimizes the mean’s squared error sum between each sample in a category. The number of categories here is defined as the number of Gaussian components in GMM. We initialize EM by *K*-means clustering.

The objective function of K-means clustering is defined as:

(11) $$\left[J=\sum_{i=1}^{K}\left(\sum_{x_{i}\in G_{i}}d_{ij}^{2}\right)=\sum_{i=1}^{K}\sum_{j=1}^{n}\mu_{ij}d_{ij}^{2}\right]$$

The objective function of category *i* is expressed as:

(12) $$\left[J_{i}=\sum_{x_{i}\in G_{i}}d_{ij}^{2}\right]$$

where *d_ij_* is the distance between category *i*’s cluster center *m_i_* and *j*-th value *x_j_*. It is expressed by Euclidean distance. µ*_ij_* is defined as:

(13) $$\left[\mu_{ij}=\left\{ \begin{array}{l} 1,\left|x_{j}-m_{i}\right|\leq \left|x_{j}-m_{K}\right|,\;i\neq K\\ 0,\;\;\;\;\;\;\;\;\;\;\;\;\;\;\;\;\;\;\;\;\;\;\;\;\;\;\;\;\;\;\;\;\;\;\;\;\;\;\;\;\;\;\;else \end{array}\right.\right]$$

*x_j_* belongs to category *i* when µ*_ij_* = 1, and otherwise, µ*_ij_* = 0. µ*_ij_* satisfies the following constraint:

(14) $$\left[\sum_{i=1}^{K}\mu_{ij}\text{=}1,j\in \left[1,n\right]\right]$$

The general process of running K-means clustering is moving in the direction of where it decreases the objective function’s value, and finally minimizes objective function to get an optimized clustering result. One observation is that the setting of initial values plays an important role in increasing the precision of EM. Thus, in this paper, we first use K-means clustering to get the initial values, and then combine particle swarm optimization (PSO) and K-means clustering to optimize the initial values for getting better estimated results. PSO is one of the evolutionary algorithms. It starts from a random solution and seeks the approximate optimal solution via continual iteration. In each round of iteration, it evaluates the quality of a solution by a fitness function, and finds the global optimal solution by following the current optimal value. PSO has the advantages of easy implementation, rapid convergence, and high precision, so we use PSO to optimize the initial values that obtained from K-means clustering. The parameters need to be optimized including three initial values: mixed coefficient, mean vector and covariance matrix. Thus, we set a three-dimensions solution space in particle swarm, and set the number of particle swarms to *m*, and the fitness function is:

(15) $$\left[fitness=F\left(x;\theta \right)=\prod_{k=1}^{K}w_{k}g\left(x;P_{k},\sigma_{k}\right)\right]$$

The combination of PSO and K-means clustering performs well on the issue of optimizing initial parameters of GMM. Moreover, via K-means clustering and PSO, we can distinguish the LOS estimated values and the varying intensity of erroneous NLOS measurements. The RSSI-based distance measurement model indicates that comparing to the signal intensity in LOS scenario, the RSSI measurement in NLOS scenario contains negative deviation. Thus, we select the RSSI signal with the highest intensity as the estimated result.

## 4. Design of RSSI-Based GMDS

Previous metric MDS localization algorithms for WSNs’ localization mostly rely on distance information to obtain their relative coordinates between nodes within a low-dimensions space. However, training a transformation model from RSSI value to distance needs large *a priori* data sets, meanwhile, the static transformation model cannot adapt to dynamic wireless signal propagation models due to continuous parameter change. Therefore, if we directly use estimated signal intensity values for localization instead of transforming it to distance, a complex transformation process be avoided, while at the same time preventing the involvement of transformation error caused by inaccurate parameters in the wireless signal propagation model. Hence, in this paper, we use a non-metric MDS technique for localization. Non-metric MDS does not require strict relationships of the dissimilarity and distance between entities, it only needs to satisfy monotonous sequence hierarchy without quantitative expressions.

### 4.1. Classical MDS (CMDS)

Let *p_ij_* be the dissimilarity between node *i* and node *j* (can also be expressed as distance information between nodes, or closeness). We can construct a dissimilarity matrix *P*, whose single element is dissimilarity *p_ij_*. If there are *n* objects, then the dimension of a dissimilarity matrix *P* is *n* × *n*. In the *m*-dimension space, the Euclidean distance between two points $X_{i}=(x_{i1},x_{i2},\cdots x_{im})$ and $X_{j}=(x_{j1},x_{j2},\cdots x_{jm})$ is expressed as:

(16) $$\left[d_{ij}=\sqrt{\sum_{k=1}^{m}{\left(x_{ik}-x_{jk}\right)}^{2}}=\left\|x_{i}-x_{j}\right\|\right]$$

The classical MDS constructs a nodes’ relative coordinates map in a multi-dimensional space, and making the dissimilarity *p_ij_* between objects close to the relative distance *d_ij_* between nodes. The closeness is expressed by *Stress*:

(17) $$\left[Stress=\sum_{i,j,i\neq j}{\left(p_{ij}-d_{ij}\right)}^{2}\right]$$

The MDS aims to minimize *Stress*. It finds the relative coordinates in a multi-dimension space as follows:

(1) Square-centralize the *n × n* dissimilarity matrix *P* of the *n* objects:
  (18) $$\left[\begin{array}{l} -\frac{1}{2}(p_{ij}^{2}-\frac{1}{n}\sum_{j=1}^{n}p_{ij}^{2}-\frac{1}{n}\sum_{i=1}^{n}p_{ij}^{2}+\frac{1}{n^{2}}\sum_{i=1}^{n}\sum_{j=1}^{n}p_{ij}^{2})=\sum_{k=1}^{m}x_{ik}x_{jk}\\ b_{ij}=-\frac{1}{2}(p_{ij}^{2}-\frac{1}{n}\sum_{j=1}^{n}p_{ij}^{2}-\frac{1}{n}\sum_{i=1}^{n}p_{ij}^{2}+\frac{1}{n^{2}}\sum_{i=1}^{n}\sum_{j=1}^{n}p_{ij}^{2})\\ B={\left[b_{ij}\right]}_{n\times n} \end{array}\right]$$
(2) Perform SVD (Singular Value Decomposition) on matrix *B*, or *B = AVA*, and then the relative coordinates of all points in a multi-dimension space are X=VA12;
(3) Top *r*(*r* ≤ *m*) columns in *X* are the solution of MDS in a low-dimension space. For example, if *r = 2*, we can obtain the relative coordinates of all points in a 2D space. In this paper, we only focus on 2-Dimensional space, but the advantage of our algorithm is it can be easily extended to 3D environment by simply set *r* = *3*.

### 4.2. GMM-Based MDS (GMDS)

In practice, it is very hard to draw an ideal distribution of P(r) from limited data, because the propagation of wireless signals is susceptible to affect by reflection, obstacles and multipath propagation, which impair the consistency of conversion between RSSI and distance. One of the observations is that the RSSI at the same distance fluctuates largely in indoor space. Therefore, when the RSSI-based localization algorithms transform the signal intensity to distance value, it inevitably involves errors, which will reduce the precision and stability.

The relationship between RSSI and distance is a monotonous function. A smaller distance means a larger RSSI value, and vice versa. This monotonous relationship satisfies the requirement of non-metric MDS’s constrain about dissimilarity data. In this paper, by combining GMM estimated RSSI values with the MDS method, we propose an algorithm for localization of indoor targets: GMDS. This algorithm includes three parts: (1) estimate RSSI values based on GMM, and construct a dissimilarity matrix *P* in MDS; (2) use MDS to compute a node’s relative coordinates; (3) transform the relative coordinates to actual coordinates.

GMDS proceeds as follows:

Step l. Construct a sparse matrix $\left[r_{ij}\right]$ based on the RSSI estimated values of GMM, where $\left[r_{ij}\right]$ is the RSSI value between nodes *i* and *j*.

Step 2. Invert the sign of every element in matrix $\left[r_{ij}\right]$ to yield a dissimilarity matrix $\left[p_{ij}\right]$, namely, $\left[p_{ij}]=[0]-[r_{ij}\right]$. Traditional MDS localization algorithm use distance to build the dissimilarity matrix, in our algorithm, we use RSSI to build the dissimilarity matrix. However, RSSI and distance have an inversely proportional relation, or in other words, a smaller distance means a larger RSSI value, and vice versa. So we invert this inversely proportional relation by simply invert the sign of the values.

Step 3. Run non-metric MDS on dissimilarity matrix $\left[p_{ij}\right]$ to form a relative coordinates map for each network’s nodes.

Non-metric MDS is an iterative process and can be described as follows:

(1) Use classical MDS to initialize all nodes’ coordinates $\left(x_{i}^{0},y_{i}^{0}\right)$ and assign initial estimated coordinates $\left(x_{i}^{0},y_{i}^{0}\right)$ to all nodes.
(2) Compute the Euclidean distance $d_{ij}$ for each node pair.
(3) Perform Pair Adjacent Violator (PAV) stepwise monotonic regression on dissimilarity matrices $\left[p_{ij}\right]$ and $\left[{d_{ij}}^{k}\right]$ to get matrix $\left[{{\hat{d}}_{ij}}^{k}\right]$.
  For any *i*, *j*, *u* and *v*, if $p_{ij}<p_{uv}$, $d_{ij}^{k}>d_{uv}^{k}$, then ${\hat{d}}_{ij}^{k}={\hat{d}}_{uv}^{k}=(d_{ij}^{k}+d_{uv}^{k})/2$; if $d_{ij}^{k}<d_{uv}^{k}$, then ${\hat{d}}_{ij}^{k}=d_{ij}^{k}$ and ${\hat{d}}_{uv}^{k}=d_{uv}^{k}$.
(4) *k* increases by 1, the new coordinates $\left({x_{i}}^{k},{y_{i}}^{k}\right)$ are updated as follows:
  (19) $$\left[\begin{array}{l} {x_{i}}^{k}={x_{i}}^{k-1}+\frac{\alpha }{n-1}\sum_{j\in M,j\neq i}\left(1-\frac{{{\hat{d}}_{ij}}^{k}}{{d_{ij}}^{k}}\right)\left({x_{j}}^{k-1}-{x_{i}}^{k-1}\right)\\ {y_{i}}^{k}={y_{i}}^{k-1}+\frac{\alpha }{n-1}\sum_{j\in M,j\neq i}\left(1-\frac{{{\hat{d}}_{ij}}^{k}}{{d_{ij}}^{k}}\right)\left({y_{j}}^{k-1}-{y_{i}}^{k-1}\right) \end{array}\right]$$
  where *n* is the total number of nodes to be localized; α is the step length of each iteration, we set α = 0.2 after carrying out simulations in Matlab, the detailed process for selecting a better α is as follows: firstly, build a dissimilarity matrix of RSSI and list 10 candidate values of α: {0.1, 0.2, 0.3, 0.4, 0.5, 0.6, 0.7, 0.8, 0.9, 1.0}; then by running the GMDS algorithm, the average positioning error under each α value can be obtained; finally, according to the results of experimental simulation, we find that the appropriate α value interval is [0.2, 0.5]. We simply select 0.2 as α value in our paper.
(5) Update Euclidean distance of each node pair.
(6) Compute *Stress* on the basis of Equation (17).
(7) If *Stress* below the threshold, then the algorithm ends; otherwise, go to (3).

Step 4. Based on the known anchor nodes, transform the relative coordinates map to an absolute coordinate map.

It is implemented by matrix transformation. To obtain the actual position, we adopt a plane four-parameter model to transform coordinates from the actual coordinates of anchor node to that of the target node. The four-parameter coordinate transformation model is:

(20) $$\left[\left[x_{T}\;\;\;\;\;\;\;y_{T}]=[x_{R}\;\;\;\;\;\;\;y_{R}]•\left[\begin{array}{l} (1+m)\cos\beta \;\;\;\;\;\;\;\;\;\;\;\;\;\;\;(1+m)\sin\beta \;\\ -\;(1+m)\sin\beta \;\;\;\;\;\;\;\;\;(1+m)\cos\beta \end{array}\right]+[\Delta x\;\;\;\;\;\;\Delta y\right]\right]$$

where $\left[x_{T}\;\;\;\;\;\;\;y_{T}\right]$ is the true coordinates after transformation; $\left[x_{R}\;\;\;\;\;\;\;y_{R}\right]$ is the relative coordinates in 2D space; *m*, β, Δ*x*, Δ*y* are parameters. We first estimate the optimal parameters that can transform the anchor node’s relative coordinates to its actual coordinates, and then transform all nodes’ relative coordinates to actual coordinates by using the above equation.

## 5. Experimental Verification and Effect Analysis

### 5.1. Construction of Experiment Platform

**Figure 1:**
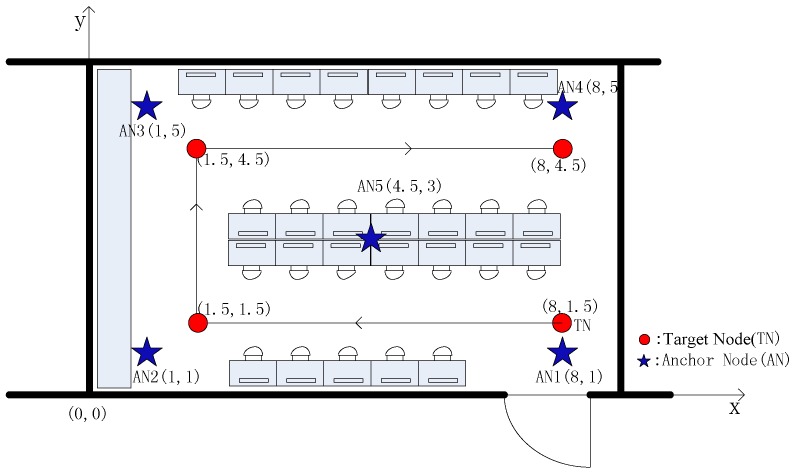
Environment deployment.

We construct an experimental environment at Lab 104 of the technical building of Northeastern University to conduct experimental verification about the feasibility and effectiveness of our proposed Gaussian mixed model based non-metric multidimensional localization algorithm. The entire experimental environment is arranged as Figure 1. The range of this indoor environment is 10 m × 6 m. The coordinates of five anchor nodes are AN1 (8, 1), AN2 (1, 1), AN3 (1, 5), AN4 (8, 5) and AN5 (4.5, 3), respectively. The target’s motion track starts from the point (8, 1.5). After proceeding 6.5 m ahead, the target turns right and proceeds 3 m ahead, before continuing to turn right and proceeding 6.5 m ahead. The target covers a track of 16 m in total length. A localization experiment is made every 0.5 m the target node moves forward, so there are 33 localization experiments in total. At each localization point, the staff simulates non-line-of-sight error by moving backwards and forwards between the anchor node and the target node to collect a group of RSSI data during this process.

**Figure 2:**
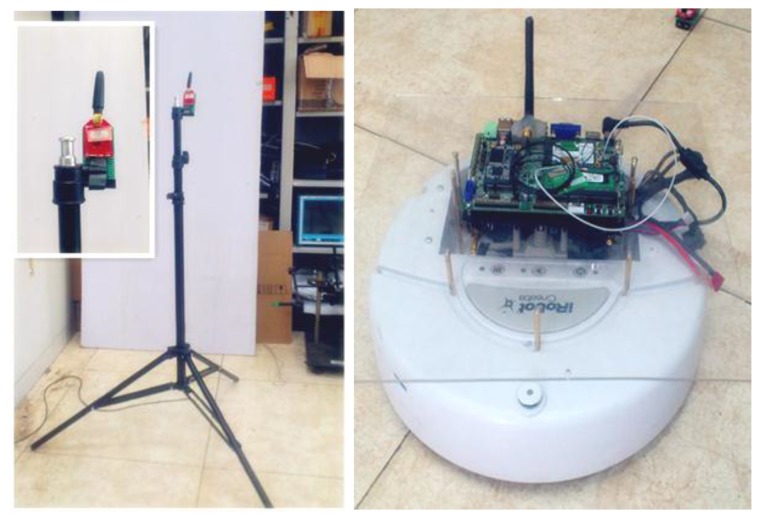
ZigBee nodes deployment.

In the experiment, ZigBee nodes are adopted as both the anchor nodes and target node. The anchor nodes are placed on a tripod with height of 1.6 m. The target node is carried by the mobile iRobot, as shown in Figure 2.

### 5.2. GMM Estimation

**Figure 3:**
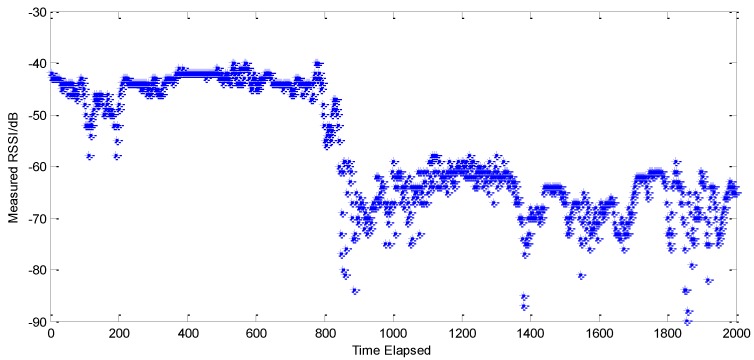
RSSI measurements.

Under the assumption that the signals have uniform transmitting powers, localization algorithms can compute distance between nodes through a signal conversion model based on RSSI. One of the advantages of our method is that we don’t need a training set to construct a signal conversion model in our experiments. We just need to collect the RSSI measured values between nodes, transform them into RSSI estimated values via a GMM, and then use estimated values for localization directly. In this way, we first need to obtain RSSI data between nodes. For example, at the localization point 33, the anchor node AN1’s initial measured data are shown in Figure 3, which reveals that the measured data are influenced by NLOS drastically and have considerable fluctuation. Therefore, if one directly uses an average RSSI value obtained by measurement for localization must cause a big error. This state of affairs becomes even worse if RSSI signal dissemination model is utilized to perform a distance conversion over the measured means.

Our algorithm adopts GMM to estimate the initial measured data of RSSI. Table 1 shows the GMM estimation results, sample means and reference RSSI values of AN1 at the localization point 33.

**Table 1:** Experimental result data.

Algorithm	RSSI (dB)
GMM-RSSI	−43.6757
Sample mean	−57.0877
Reference	−42.9588

**Figure 4:**
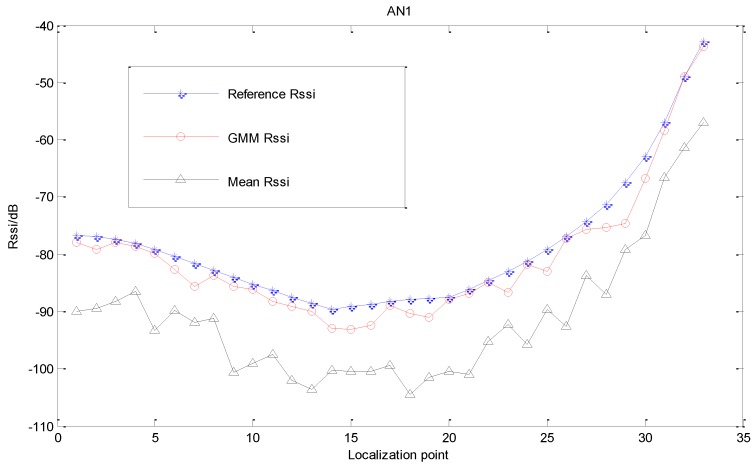
Result of RSSI estimation.

With the same measurement, the estimation results of all localization points of AN1 are shown in Figure 4. It can be seen from the experimental result that the Gaussian Mixed Model can effectively reduce the error of measured values and get a more accurate RSSI estimated values under NLOS environment.

**Figure 5:**
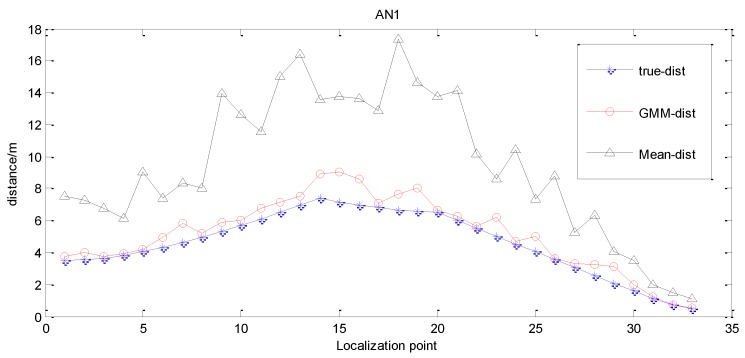
Result of distance estimation.

After obtaining relatively accurate estimated RSSI values, the distances between nodes can be computed based on the RSSI distance computation model. The result diagram of distance estimation of the anchor node AN1 is shown in Figure 5. Compared to the approach of utilizing the measured means to estimate distances directly, the estimation by GMM gets more accurate distance estimation.

From the above analysis, it can be known that the GMM estimation proposed in this paper can effectively reduce the error caused by NLOS on measured RSSI data, and enhancing the accuracy of target localization.

### 5.3. Effectiveness of the GMDS Localization Algorithm

To demonstrate the effectiveness of the algorithm, a comparative experiment is conducted between the GMDS localization algorithm proposed in this paper, and least square method (LS) along with two state-of-the-art algorithms: the residual weighting algorithm (Rwgh) [5,6] and classic multidimensional scaling algorithm (CMDS) [28].

**Figure 6:**
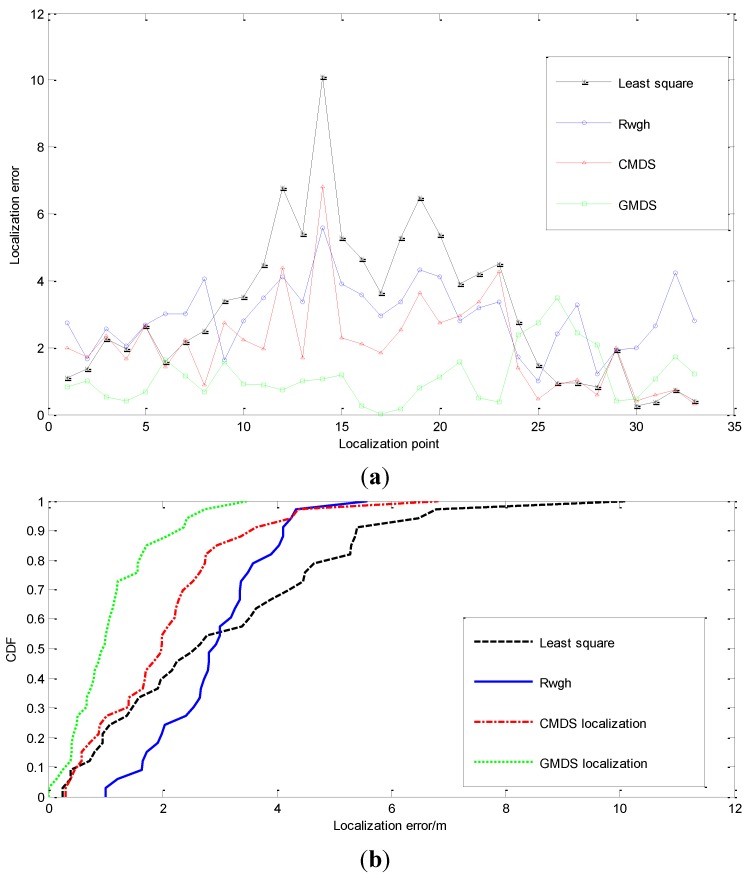
Performance of the tracking algorithms when $N_{nlos}=1$. (**a**) Localization error when $N_{nlos}=1$; (**b**) Diagram of cumulative error distribution when $N_{nlos}=1$.

**Figure 7:**
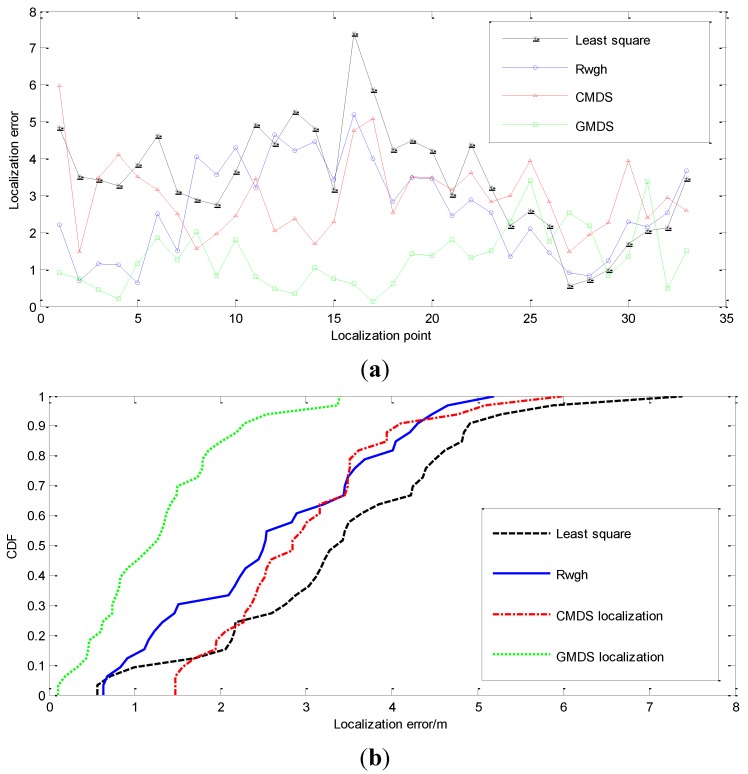
Performance of the tracking algorithms when $N_{nlos}=2$. (**a**) Localization error when $N_{nlos}=2$; (**b**) Diagram of cumulative error distribution when $N_{nlos}=2$.

**Figure 8:**
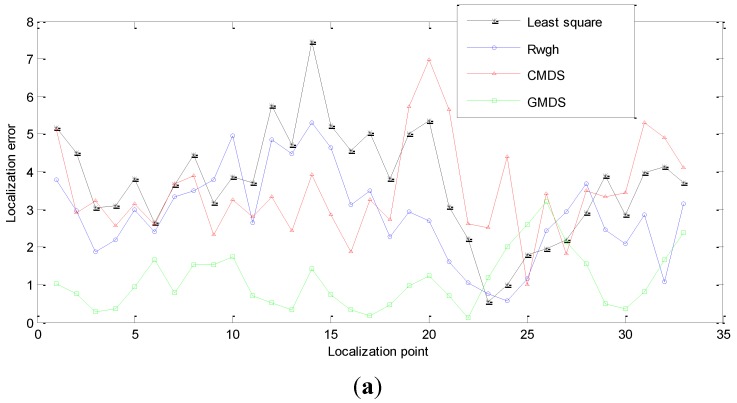

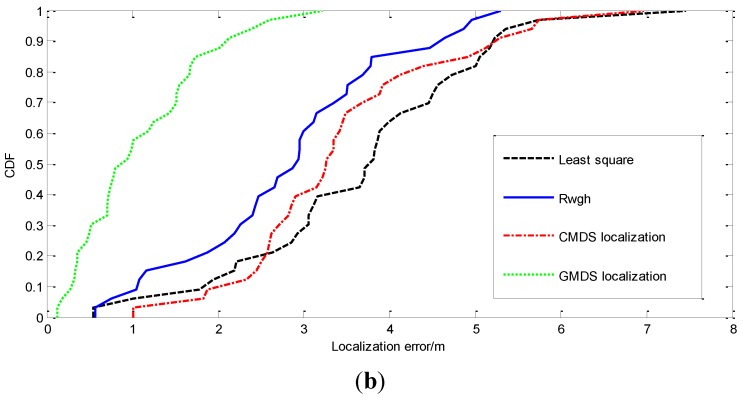
Performance of the tracking algorithms when $N_{nlos}=3$. (**a**) Errors of localization when $N_{nlos}=3$; (**b**) Diagram of cumulative error distribution when $N_{nlos}=3$.

We conduct a comparative experiment about the effectiveness of localization under different numbers of NLOS nodes. Figure 6 shows the localization result when one of the anchor nodes and targets are in the NLOS state; Figure 7 shows the localization result when the states of channel between two anchor nodes and the target are both in NLOS state; Figure 8 shows the experimental result when there are three nodes in NLOS state. The localization error of these four algorithms is shown in Figure 6a, Figure 7a and Figure 8a. Figure 6b, Figure 7b and Figure 8b renders statistics about the cumulative error distribution of localization errors. Table 2 shows the mean localization errors of these algorithms.

**Table 2:** Average errors of localization (m).

Algorithm	*N_nlos_* = 1	*N_nlos_* = 2	*N_nlos_* = 3
GMDS	1.2057	1.3028	1.3111
CMDS	2.6223	2.9836	3.4741
LS	3.2646	3.4515	3.7053
Rwgh	2.7367	2.6401	2.8519

Based on the analysis of experimental result, it can be seen that, under the NLOS state, the GMDS algorithm is able to achieve higher accuracy and effectiveness in localization and tracing. The LS, residual weighting method and classic multidimensional scaling algorithm need to convert RSSI to distance estimation by the dissemination model to implement localization estimation. The converting requires prior knowledge about conversion parameters for specific setting. While our algorithm can perform a localization estimation directly by using estimated RSSI values without being provided with prior knowledge about the setting. It is of higher applicability under different settings; meanwhile, it can achieve higher accuracy of localization under the NLOS setting.

## 6. Conclusions

In an NLOS propagation environment, a majority of RSSI-based localization algorithms rely on the signal propagation model that transforms the RSSI measurements to distances between nodes, and then uses NLOS localization algorithms to compute the target node’s coordinates and to obtain the positional information. Unfortunately, the RSSI values are susceptible to fluctuate under the influence of NLOS, the distances obtained from the propagation model often contain large errors, leading to low precision and low effectiveness in localization. This paper proposes a new algorithm GMDS, which adopts GMM to estimate the RSSI measurements and uses RSSI estimated values directly in localization. The use of MDS reduces both computation complexity and errors. The experimental verification shows that in an indoor NLOS environment, GMDS effectively reduces NLOS errors and has a high rate of effectiveness and wide applicability in localization. GMDS also shows considerable scalability when extending single NLOS to multiple NLOS.
